# Firefighters’ occupational exposure to air pollution: impact on COPD and asthma—study protocol

**DOI:** 10.1136/bmjresp-2023-001951

**Published:** 2024-11-20

**Authors:** Joana V Barbosa, Pedro T B S Branco, Maria C M Alvim-Ferraz, Fernando G Martins, Sofia I V Sousa

**Affiliations:** 1 LEPABE—Faculdade de Engenharia, Universidade do Porto, Porto, Portugal; 2 ALiCE – Associate Laboratory in Chemical Engineering, Faculty of Engineering, University of Porto, Porto, Portugal

**Keywords:** Asthma Epidemiology, COPD epidemiology

## Abstract

**Introduction:**

Firefighting continues to be among the most hazardous yet least studied occupations in terms of the impact of exposure to occupational disease. In particular, firefighters are at increased risk of adverse health effects due to exposure to significant levels of potentially harmful substances, namely carbon monoxide, particulate matter and formaldehyde, during their professional duties.

This paper reports an epidemiologic study aiming to reduce the gaps in assessing the long-term effects of air pollution exposure to forest fires’ combat on firefighters, namely regarding chronic obstructive pulmonary Disease (COPD) and asthma.

**Methods and analysis:**

Based on the implementation in an area with high forest fires (in Portugal), the study will analyse firefighters’ exposure to fire emissions by measuring air pollutants with personal exposure monitors during forest fire combat through a retrospective cohort study (exposed vs non-exposed). Moreover, based on answers to validated questionnaires and medical examinations to be performed by medical doctors, the study will assess the prevalence, incidence and exacerbation of COPD and asthma in firefighters, thus considering both short-term and long-term effects. Based on the results above referred, the study aims to evaluate the impact of exposure and inhalation dose of air pollutants during forest fires’ combat on the development of the above-referred chronic diseases. The approximate number of participants in the study will never be less than 186, guaranteeing 80% of study power (significant at a 5% level).

**Ethics and dissemination:**

The study has been approved by the Ethical Committee of Centro Hospitalar Universitário São João. The results will be published in international and national journals and conferences, allowing the results obtained to be communicated to the scientific community. Moreover, up-to-date data will be disseminated to stakeholders and decision-makers to help them decide on triggering official control measures.

**WHAT IS ALREADY KNOWN ON THIS TOPIC**

- Recent studies have shown that firefighters are exposed to hazardous air pollutants while fighting forest fires and are, therefore, at increased risk of developing respiratory symptoms or diseases.

**WHAT THIS STUDY ADDS**

- This retrospective cohort study will contribute to understanding the impact of air pollution from forest fires’ combat on the respiratory health of firefighters, namely regarding the development of asthma and COPD.

**HOW THIS STUDY MIGHT AFFECT RESEARCH, PRACTICE OR POLICY**

- This study will enable the quantification of impacts and will contribute to launching recommendations for forest firefighting management taking into account those impacts.

## Introduction

 There is an increased interest in the health impacts of forest fires because there is scientific consensus that the number and intensity of forest fires will increase due to climate change.^1^

Recent systematic reviews^1 2^ concluded that a strong association exists between exposure to forest fire smoke and respiratory morbidity, especially with chronic obstructive pulmonary disease (COPD) and asthma exacerbations. Occupational asthma is usually missed, but even so, an estimated 5–20% of new cases of adult-onset asthma can be attributed to occupational exposure.^3^ Work-related asthma, which includes occupational and work-aggravated asthma, has become one of the most prevalent occupational lung diseases.^4^ Some occupational exposures that are potential causes of occupational asthma, particularly high concentrations of air pollutants, burning of biomass fuel, environmental tobacco smoke and wood dust, have also been reported to cause COPD.^4^ COPD is one of the top three leading causes of death in the world and was responsible for 6% of deaths globally in 2012.^5^ COPD is a heterogeneous lung condition characterised by chronic respiratory symptoms (dyspnoea, cough, sputum production and/or exacerbations) due to abnormalities of the airways (bronchitis, bronchiolitis) and/or alveoli (emphysema) that cause persistent, often progressive, airflow obstruction, which is caused by significant exposure to noxious particles or gases from indoor or outdoor air pollution, with tobacco smoking being the main risk factor.^5^ Accordingly, firefighters may be at increased risk of adverse health effects due to their exposure to all kinds of potentially harmful substances during their professional duties.^6^ Forest firefighters comprise an occupational group with high exposure to biomass smoke. Studies on exposure among forest firefighters have shown that they can be exposed to significant levels of atmospheric pollutants, including carbon monoxide (CO), formaldehyde and particulate matter.^6 7^ Firefighting continues to be among the most hazardous yet least studied occupations in terms of exposures and their relationship to occupational disease.^8^ Some studies have been performed essentially on Australian and USA firefighters,^6^ but some only evaluated the respiratory health impairments before and after fire combat and others evaluated only air pollutants’ exposure without evaluating its impacts on health. Several studies were performed worldwide evaluating the impact of exposure on the respiratory health of firefighters.^9 10^ Results showed consistently a decline in forced expiratory volume in 1 s (FEV_1_) following a full season of firefighting compared with preseason values and that this value returned to baseline FEV_1_ in the postseason.^9^ Moreover, one study comparing pre-shift and post-shift values instead of preseason and postseason values reported no change in FEV_1_, suggesting that lung function decline is not an acute event but is rather associated with longer smoke exposures.^11^ However, the cumulative effect of repeated forest fire smoke injury on the lungs remains unknown.^9 10^

Portugal has consistently been one of the European countries with the highest burnt area.^2^ The most affected districts have been from the North and Centre, with Viseu being the district with a combined higher number of forest fires and burnt areas (2009–2015).^12^

In Portugal, only one project (concluded in 2010) assessed the impact of air pollution exposure from forest fires on the lung function of firefighters from the district of Coimbra. Results showed high exposures to air pollutants during forest fires and diminished lung function. Although an important contribution, this study did not fully cover firefighters’ exposure and did not study long-term effects, namely COPD and asthma development.^13 14^ This project aims to reduce the lacks above referred, adding to the state-of-the-art through the assessment of the long-term effects of exposure to air pollution from forest fires on firefighters, namely regarding COPD and asthma, with a cohort (exposed vs non-exposed) study, performed on Viseu District (Portugal), by: (1) fully characterising firefighters’ exposure (at fire stations and during forest fire combat), namely through the measurement of CO, sulphur dioxide (SO_2_), nitrogen oxides (NOx), particles, ozone (O_3_), total volatile organic compounds (VOC) and formaldehyde; (2) evaluating the inhalation dose of air pollutants by firefighters; (3) assessing the prevalence, incidence and exacerbation of COPD and asthma; (4) evaluating the impact of the above-referred pollutants exposure during firefighting on the development of COPD and asthma on firefighters and (5) contributing to a better supported development of preventive measures.

Globally, expected results are to: (1) evaluate air pollutants concentration to which firefighters are exposed during forest fires; (2) develop a complete exposure assessment of firefighters by including measurements at fire stations and during forest fire combat; (3) calculate the prevalence and incidence of COPD and asthma on firefighters based on medical evaluation, as well as the exacerbation of respiratory health symptoms during forest fire combat season; (4) identify pollutants that may increase the prevalence and incidence of COPD and asthma on firefighters and exacerbate respiratory symptoms. This will allow the development of strategies to (5) reduce exposure to those pollutants, aiming to reduce the incidence rate of COPD and asthma in firefighters; (6) support direct and indirect environmental education, namely through flyers’ distribution and through the participation of firefighters in the results obtained and on the strategies to reduce exposure and thus the impact on the chronic diseases above referred; and (7) report results to the scientific community and communicate results to stakeholders and decision-makers that will help decide on triggering official control measures.

## Methods and analysis

**Figure 1:**
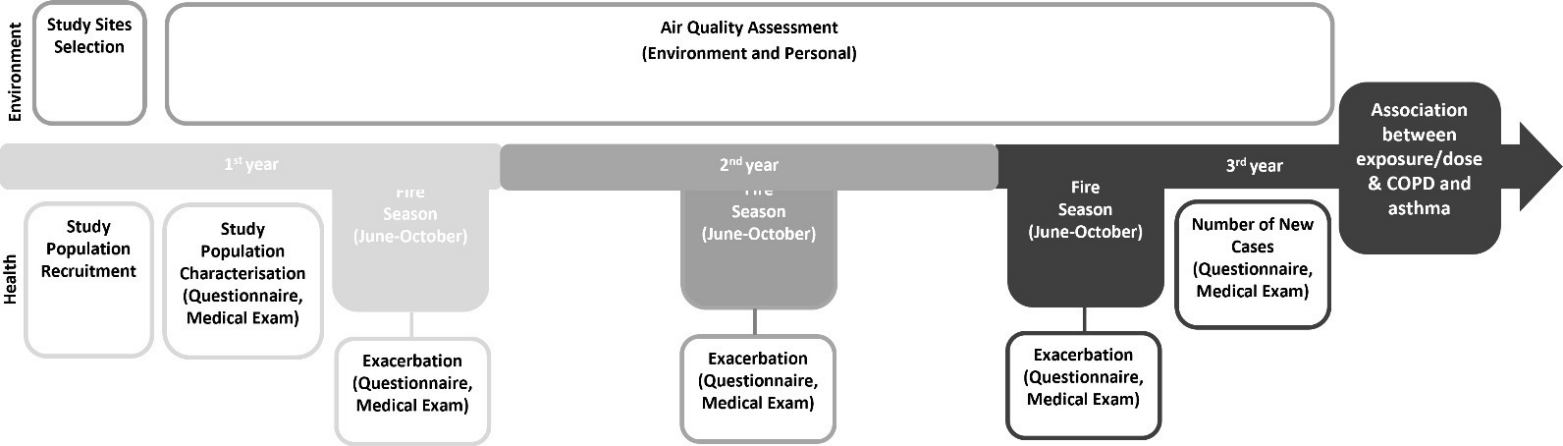
Summary representation of the methodology to be applied. COPD, chronic obstructive pulmonary disease

A cohort retrospective study will be performed in 3 years and will include six main stages as described below. Figure 1 presents a summary representation of the methodology to be applied.

### Selection of the study sites and population

At this stage, it is expected to select the sites (fire stations) in Portugal where the study will be performed and the study population, namely exposed and non-exposed. Portugal is located in the southwest of Europe and is bordered by the Atlantic Ocean in the north, west and southwest. In Portugal, the fire season usually occurs between June and October, a period when high temperatures and low relative humidity are usually recorded.

The study will be performed in the Viseu District (one of the 18 Portuguese districts), as this is the Portuguese district with the highest combined number of forest fires and burnt areas (2009–2015). According to the latest Portuguese data, in 2022, there were a total of 1292 firefighters in Viseu District distributed across 33 fire stations. Non-exposed firefighters will be those with no experience/time spent in the combat of forests or other types of fires and also firefighters who have not fought fires for more than 10 years. Exposed firefighters will be those who have fought in the last 5 years and/or will be fighting forest fires. Moreover, non-exposed firefighters with medical issues or comorbidities related to the main outcome will be excluded. Sample size calculations were performed to find the minimum number of exposed and non-exposed firefighters to enroll in the study and to detect significant differences between groups considering a threefold increase in COPD prevalence for firefighters combating forest fires (exposed). The number of participants will be 186 (93 non-exposed/93 exposed, WinPepi V.11.65, COMPARE2 package) considering a cohort study and a significance level of 5%, power of 80%, the estimated prevalence of 14.2%,^15^ continuity correction and an oversample of 5% to compensate for potential drop-off. If a low number of professional firefighters in the conditions to participate in the study agree to participate, volunteer firefighters will also be included in the studied sample (exposed and non-exposed). For volunteers, a stratification and match for the type of work will be performed in exposed and non-exposed groups to account for different exposures at the official place of work and those with occupations that could be confounders will also be excluded. Smoker participants and those influenced by environmental tobacco smoke (at least one cohabitant smoking) will be considered in the study as a covariate to analyse synergistic effects with air pollution. In the case of a low response rate (<186 participants) on the considered fire stations, more sites will be included in the study.

### Evaluation of pollutant concentrations

At this stage, it is expected to: (1) determine the concentrations of air pollutants at fire stations and during firefighting; and (2) assess sources in cases of high concentrations at fire stations.

Pollutant concentrations, namely CO, SO_2_, NOx, particles, O_3_, total VOC and formaldehyde, will be measured at fire stations and during forest fires.

At fire stations, pollutant concentrations will be measured continuously by standard methods (passively when there is no other possibility) in warm and cold months, enabling comparisons, for 2 weeks. PM concentrations will be measured using a TSI DustTrak DRX 8533 Aerosol Monitor (TSI, USA), with light scattering, operating at 3 L/min, while NO_2_, SO_2_, CO and VOC concentrations will be measured using personal monitoring equipment (MultiRAE Lite, Honeywell International), operating at 0.2 L/min. Temperature and relative humidity will also be measured.

During forest fire combat, firefighters will use personal monitors with continuous measurements. PM concentrations will be measured using a TSI Side-Pak AM520 operating at 0.2 L/min, while the same equipment and conditions used inside the fire stations will be used to measure NO_2_, SO_2_, CO and VOC concentrations. Moreover, if fewer fires than usual are recorded during the study period, personal monitoring exposure will be performed on prescribed fires.

An assessment of the conditions of the fire stations will be performed to understand possible sources of air pollution and to act on mitigating potential high levels.

### Assessment of exposure and inhalation doses

At this stage, it is expected to assess the detailed exposure of firefighters and calculate the respective dose inhalation.

Exposure models will be developed based on a microenvironmental approach combining time-activity patterns (TAP) and pollutant concentrations in each microenvironment measured. TAP will be obtained from a participant-reported daily diary to determine the considered microenvironment (home indoor and outdoor, work indoor and outdoor, in transport and other) and activities and their duration. Inhalation doses will be calculated based on exposure and inhalation rates and body mass.

### Evaluation of COPD and asthma prevalence, incidence and exacerbation

At this stage, it is expected to (1) assess COPD and asthmatic symptoms among exposed and non-exposed firefighters and gather information about firefighters’ background; (2) evaluate the prevalence and incidence of COPD and asthmatic firefighters at the selected sites and the short-term effects of forest fire combat exposure; and (3) evaluate the exacerbation of COPD and asthmatic symptoms of participants with COPD and/or asthma at the selected sites.

Prevalence will be evaluated through a questionnaire and medical examination assessment at the beginning of the study. The incidence will be evaluated through a questionnaire and medical examination at the end of the 3-year study, through comparison with the data obtained at the beginning of the study. Exacerbation of symptoms will be evaluated through a specific questionnaire targeting respiratory symptoms after firefighting and a medical examination immediately after fighting forest fires during the study period. The main details on the questionnaires and on the medical examinations to be performed are described in the following subsections.

#### Assessment of COPD and asthmatic symptoms

Participants will be asked to sign a participation consent according to the Helsinki Declaration developed by the World Medical Association. Validated questionnaires (based on those used in the European Community Respiratory Health Survey) will be filled in by the participants (exposed and non-exposed) who agreed to participate, at the beginning and end of the study, including questions concerning gender, age, weight, type of firefighters, number of years as a firefighter, participation in forest fires, education, professional occupation, socio-economic status, tobacco smoke, food habits, allergies, amount of time spent indoor and outdoor, home conditions (like power source to cook, heat source, use of fireplaces, floor coating), medical history (such as stroke, myocardial infarction), including comorbidities (such as cholesterol, diabetes, hypertension, heart disease) and respiratory health symptoms and diseases. The questions related to respiratory symptoms and diseases will focus mainly on whether there are any previously diagnosed diseases and the use of medication, allergy-related, family history and specific respiratory symptoms such as cough, sputum, wheeze and shortness of breath. Filled questionnaires will be validated by medical doctors.

#### Medical examinations

Participants will be examined by medical doctors through spirometric tests with reversibility tests to confirm COPD and/or asthmatic symptoms. Medical examinations will be performed at the beginning of the study (after questionnaire application), before and after the fire seasons when forest fires occur in each of the 3 years of study and near the end of the study to evaluate the number of new cases. Based on the spirometry results, participants will be considered with COPD when in the presence of a post-bronchodilator FEV_1_/FVC<70% and asthmatic when: (1) the percentage of FEV_1_/FVC is less than 75–80%; and (2) the change in FEV_1_ pre and post medication is higher than 12% and higher than 200 mL than the baseline (as defined by the American Thoracic Society and European Respiratory Society).

Spirometry will be performed using a Vitalograph ALPHA Track (Vitalograph, UK) in one specific room of a fire station where medical doctors will bring the necessary equipment.

#### Evaluation of COPD and asthma exacerbation

Questionnaires will be distributed to the participants with confirmed COPD and/or asthma to assess acute respiratory symptoms with the aim of understanding if the forest fire combat has an influence on the aggravation of symptoms. Questions concerning the number of forest fires they have participated in the current season, the existence of respiratory symptoms and/or physical fatigue after fighting and how long it takes to recover, whether they use personal protective equipment (PPE) and specific questions related to the equipment cleaning will be included in this questionnaire.

### Impact of exposure/inhalation dose during fire combat on COPD and asthma

At this stage, it is expected to evaluate the impact of exposure and inhalation doses of air pollution during forest fires on COPD and asthma.

Results from previous studies (INAIRCHILD project) showed that it is not only the exposure per se but the inhaled dose that may contribute to asthma development; dose depends not only on concentrations and time of exposure but also on the type and duration of the activities. Thus, this project will assess both the impact of exposure and the inhalation dose. Exposure will be obtained by combining time patterns and pollutant concentrations measured at fire stations and forest fires. On the contrary, the inhalation dose will be estimated based on the time-averaged exposure, the inhalation rate (obtained from the US EPA approach) and the body weight of the firefighters (obtained from the questionnaires).

### Data analysis

Prevalence rates will be calculated as the ratio between the number of individuals who have the disease and the total number of participants. Incidence rates will be estimated as the number of new cases during the study period. Exacerbation will be calculated as the number of individuals with exacerbation of symptoms after firefighting and the total number of individuals that went to combat the fire. Daily exposures to indoor air pollutants in the fire stations and during firefighting and correspondent inhaled doses will be estimated for each of the participants (N=186). The characteristics of health outcomes, exposures and inhaled doses will be expressed using descriptive statistics. Continuous variables weight, height and age will be transformed into categorical variables. The respiratory health outcomes will be binary variables, and logistic regression models will be used to assess the impact of both exposure and inhaled dose of all pollutants individually (univariate analysis) and in association (multivariate analysis) on COPD and asthma development, exposure–response and dose–response, respectively, in both exposed and non-exposed.

### Ethics and dissemination

The study has been approved by the Ethical Committee of Centro Hospitalar Universitário São João (199/99). The use of human data will be performed in accordance with the Declaration of Helsinki and Portuguese legislation regarding data protection. Informed consent to participate will be obtained from the participants.

In order to help local communities better understand pollution and its sources and contribute to developing preventive measures, communication of the results to local communities and responsible entities for air pollution management will be performed as many times as possible. The results will be disseminated to the scientific community through papers’ publication in national and international journals and through a final conference/seminar. Based on the results obtained, the team will understand the dynamics that exist during firefighting, such as staff rotation and/or rest breaks to reduce the risk of prolonged exposure, the use of PPE and its impacts. With the results obtained, the team will develop strategies to reduce exposure to pollutants and communicate them to the Viseu Sub-Regional Command and to the National Authority for Emergency and Civil Protection.

### Patient and public involvement

The involvement of the public will be performed after signed consent. Please refer to the Methods and Analysis section.

## Discussion

Based on the above described, the expected results are the following:

- Find out the concentration of air pollutants to which firefighters are exposed during forest fire combat.
- Develop a complete exposure assessment of firefighters by including measurements at fire stations and the distribution of time-activity diaries for firefighters to fill out.
- Calculate the prevalence and incidence of COPD and asthma among firefighters, as well as the exacerbation of respiratory health symptoms during forest fire season.
- Understand if there are synergistic effects between smoking or being exposed to environmental tobacco smoke and air pollution from forest fire combat that can affect the development and exacerbation of COPD and asthma in firefighters.
- Identify pollutants that eventually contribute to the development and exacerbation of COPD and asthma in firefighters, considering confounding effects. This will allow the development of strategies to reduce exposure to those pollutants, as the number of hours exposed, aiming for the reduction of the incidence rate of COPD and asthma in firefighters.
- Directly and indirectly support environmental education, namely through flyers’ distribution and through the participation of firefighters on the results obtained and on the strategies to reduce exposure and thus the impact on the chronic diseases above referred to. The participant’s awareness is considered one key aspect.
- Publish in international and national journals and conferences allowing the reporting of the results obtained to the scientific community. Also, urgent dissemination of up-to-date data will be performed to stakeholders and decision makers that will help decide on triggering official control measures.
